# Dry Needling Versus Manual Therapy for Mechanical Neck Pain: A Target Trial Emulation Using Electronic Health Records

**DOI:** 10.1155/prm/3221855

**Published:** 2026-08-28

**Authors:** Wenjun Zhang, Xin Zhang, Lihua Huang, Shengdi Lu, Lei Yu

**Affiliations:** ^1^ Department of Orthopedics, Zhujiajiao People’s Hospital, Shanghai, China; ^2^ Department of Rehabilitation Medicine, Shanghai Yangzhi Rehabilitation Hospital (Shanghai Sunshine Rehabilitation Center), Shanghai, China; ^3^ Department of Rehabilitation Medicine, Shanghai Sixth People’s Hospital, Shanghai, China, 6thhosp.com; ^4^ Pennington Biomedical Research Center, Baton Rouge, Louisiana, USA, lsu.edu; ^5^ Department of Healthcare Informatics, Shanghai Sixth People’s Hospital, Shanghai, China, 6thhosp.com

**Keywords:** comparative effectiveness, dry needling, manual therapy, neck pain, target trial emulation

## Abstract

**Objective:**

To estimate the effect of dry needling versus manual therapy on disability in mechanical neck pain by emulating a target trial using electronic health record data from three tertiary hospitals in China.

**Design:**

This target trial emulation used a new‐user, active‐comparator design with alignment at time zero. Patients were new users of dry needling plus exercise (*n* = 1995) or manual therapy plus exercise (*n* = 3898). The primary outcome was the Neck Disability Index (NDI) at 3 months. Effects were estimated by inverse probability of treatment weighting (intention‐to‐treat–analogue) and clone‐censor‐weighting (per‐protocol).

**Results:**

Among 5893 patients, dry needling initiators were more severe at baseline (maximum standardised mean difference, 0.72), consistent with confounding by indication. The unadjusted 3‐month NDI difference (dry needling minus manual therapy) was 10.1 points (95% CI, 9.4–10.7). After weighting, the difference was 5.7 points (95% CI, 5.0–6.5), exceeding the minimal clinically important difference of 5.5. The per‐protocol difference was 5.7 (95% CI, 4.9–6.4).

**Conclusion:**

Under the assumptions of the target trial emulation, manual therapy was associated with greater improvement in disability than dry needling, consistent with the index trial. The unadjusted analysis overstated the association, underlining the value of confounding adjustment. Residual confounding from unmeasured factors cannot be excluded.

**Summary**
• What is Known
◦ Mechanical neck pain is common and disabling. Dry needling and manual therapy are both widely used, but head‐to‐head evidence is limited, and a single randomised trial favoured manual therapy.
• What is New
◦ Using a target trial emulation of electronic health record data from Chinese hospitals, manual therapy was more effective than dry needling, matching the randomised trial. A naive unadjusted comparison was biased by confounding. The study shows how routinely collected data can support causal comparisons of rehabilitation treatments.

## 1. Background

Neck pain is one of the most common musculoskeletal complaints worldwide. It affects hundreds of millions of people and ranks among the leading causes of years lived with disability [1]. The burden is rising in China, where prevalent cases have grown steadily over the past 3 decades [2]. Most neck pain is mechanical and nonspecific. It tends to recur, and many patients develop persistent symptoms [3].

Conservative care is the foundation of treatment. Clinical guidelines recommend exercise alongside manual techniques and other physical therapies [4]. Two active treatments are widely used. Dry needling targets myofascial trigger points with a thin filament needle, and manual therapy uses joint mobilisation and manipulation. Both are common in routine rehabilitation, yet the evidence for each is limited. Trials of dry needling are mostly small and short term [5, 6]; recent systematic reviews report short‐term reductions in pain and disability but limited or inconsistent effects on cervical range of motion, with generally low certainty of evidence [7, 8]. For manual therapy, systematic reviews and meta‐analyses similarly report small, mainly short‐term benefits of low to very low certainty [9–11], and a network meta‐analysis of 101 trials found multimodal treatment most effective for pain and disability and manipulation most effective for range of motion [12]; benefits have also been reported for manual therapy combined with exercise in chronic neck pain [13]. High‐quality head‐to‐head comparisons of dry needling and manual therapy remain scarce, and no 2024–2025 systematic review directly compares the two.

The choice between these treatments is therefore unclear. The problem is sharper in China. Dry needling overlaps with traditional acupuncture in both concept and practice, and the two are often recorded under the same billing categories [8]. Acupuncture and manual therapies are also mainstream choices for musculoskeletal pain among Chinese patients [9]. A direct comparison in this setting would help clinicians and patients.

One randomised trial has compared these treatments directly. Pandya and colleagues randomised 78 adults with mechanical neck pain to dry needling plus exercise or manual therapy plus exercise [12]. Manual therapy was more effective for pain and disability in the short and intermediate terms. The trial was small and conducted in a single centre in the United States. Whether the finding holds in routine Chinese care is unknown. A large pragmatic trial would be costly and slow.

Routinely collected electronic health record (EHR) data offer an alternative. When analysed with the target trial emulation framework, such data can support causal comparisons [10, 11]. The framework specifies a hypothetical trial and then emulates it, which reduces common biases. We applied this approach and reported it using the Transparent Reporting of Observational Studies Emulating a Target Trial (TARGET) guideline [13].

We aimed to emulate the Pandya trial using EHR data from three tertiary hospitals in China. Our objective was to estimate the effect of dry needling versus manual therapy on neck disability and pain. We examined both the effect of starting a treatment and the effect of receiving it as intended. We hypothesised that manual therapy would be more effective than dry needling, in keeping with the index trial. We further expected that simple unadjusted comparisons would be biased, because sicker patients are channelled to one treatment, and that this bias could be reduced by appropriate methods.

## 2. Methods

### 2.1. Study Design and Population

We emulated a target trial comparing dry needling with manual therapy for mechanical neck pain, following the target trial emulation framework and the TARGET guideline [10, 13, 14]. The protocol of the index trial guided the specification [12]. Table 1 sets out each protocol component and its emulation; detailed operationalisation is given in the Supplementary Material. We used EHR data from three tertiary hospitals in China (January 2019 to December 2024). Because the data are observational, eligibility, treatment assignment and start of follow‐up were aligned at a single time zero to avoid immortal time bias and selection bias [15, 16]. Reporting follows STROBE [17].

**TABLE 1:** Summary of the specification of the target trial and its emulation using observational electronic health record (EHR) data.

Protocol component	Target trial specification	Emulation using observational EHR data
Eligibility criteria	Adults (> 18 years) with mechanical neck pain; baseline Numeric Pain Rating Scale (NPRS) ≥ 2 and Neck Disability Index (NDI) ≥ 10/50 (≥ 20%). Exclusions: cervical/thoracic surgery; neurological signs or nerve‐root compression/myelopathy; whiplash within 6 weeks; red flags (malignancy, infection, fracture); contraindications to dry needling or manual therapy.	The same criteria were operationalised from EHR at time zero using ICD‐10 (National Clinical Version 2.0) diagnosis codes, structured fields and free‐text notes (supporting table S2 and code lists S8–S10). All criteria ascertained on or before time zero; no postbaseline information used. New‐user requirement: no dry needling or manual therapy for neck pain in the prior 12 months.
Treatment strategies	(1) Dry needling + usual therapeutic exercise; (2) manual therapy (mobilisation/manipulation) + usual therapeutic exercise. Index trial dose: 7 sessions over a maximum of 6 weeks.	Strategy assigned by the first qualifying dry needling or manual therapy session identified from national rehabilitation/TCM procedure‐charge items and free‐text notes (supporting table S9), distinguishing dry needling from acupuncture and manual therapy from tuina/massage; validated by dual chart review.
Assignment procedures	Random assignment to one strategy; participants aware of allocation.	Classification (not randomisation) into the strategy compatible with the first qualifying session at time zero. Confounding by indication addressed by IPTW on measured baseline covariates (emulating randomisation conditional on covariates).
Time zero and follow‐up	Follow‐up starts at assignment and ends at the 3‐month postdischarge assessment (with interim assessments at 2 weeks and discharge).	Time zero = date of first qualifying session; eligibility, assignment and start of follow‐up are aligned at time zero to prevent immortal time and selection bias. Outcome windows: 2–3 weeks, 4–6 weeks (discharge) and 3 months. Administrative end 31 December 2024; censoring at death, loss to follow‐up (no further encounters) and, for the per‐protocol analysis, protocol deviation.
Outcomes	NDI (primary), NPRS; and (not emulable here) PSFS, GROC, FABQ, DNFET.	NDI (0–50, simplified Chinese version) and NPRS (0–10) were extracted from rehabilitation patient‐reported‐outcome tables at each window. PSFS, GROC, FABQ and DNFET are not recorded in the EHR and cannot be emulated (limitation).
Causal contrasts	Intention‐to‐treat effect (effect of assignment) and per‐protocol effect (effect of receiving the assigned strategy per protocol).	ITT‐analogue: effect of initiation, estimated by stabilised IPTW. Per‐protocol: effect of receiving the assigned strategy with an adequate dose (≥ 5 of 7 sessions within 42 days, 14‐day grace, no switching/discontinuation), estimated by clone‐censor‐weighting with inverse‐probability‐of‐censoring weights. Reported as mean differences (absolute) and proportions achieving the minimal clinically important difference (relative).
Identifying assumptions	Exchangeability, positivity and well‐defined interventions; for per‐protocol, no unmeasured time‐varying confounding of adherence.	Conditional exchangeability given measured baseline covariates (no unmeasured confounding); positivity assessed by propensity‐score overlap; consistency assumed. Robustness assessed by E‐values, negative‐control outcome and sensitivity analyses (supporting tables S5–S7).
Statistical analysis	Mixed‐model repeated‐measures analysis; intention‐to‐treat.	Propensity score by logistic regression; stabilised IPTW truncated at 1st/99th percentiles; weighted linear models with robust (HC1) variance for ITT‐analogue; clone‐censor‐weight with bootstrap variance for per‐protocol; missing data by multiple imputation (chained equations). SMD < 0.10 defined balance.

*Note:* The target trial is the randomised controlled trial of Pandya et al. (J Orthop Sports Phys Ther 2024; 54 [4]:267–278; NCT04851067). Each protocol component is specified for the hypothetical target trial (centre column) and operationalised in the observational emulation (right column). Time zero (start of follow‐up) was aligned with the date of eligibility and treatment assignment to prevent immortal time and selection bias. The Patient‐Specific Functional Scale, global rating of change, Fear‐Avoidance Beliefs Questionnaire and deep neck flexor endurance test were measured in the index trial but are not recorded in the electronic health record and therefore could not be emulated.

Abbreviations: CCW = clone‐censor‐weight, DNFET = deep neck flexor endurance test, EHR = electronic health record, FABQ = Fear‐Avoidance Beliefs Questionnaire, GROC = global rating of change, ICD‐10 = International Classification of Diseases, 10th Revision, IPTW = inverse‐probability‐of‐treatment weighting, ITT = intention‐to‐treat, NDI = Neck Disability Index, NPRS = Numeric Pain Rating Scale, PSFS = Patient‐Specific Functional Scale, SMD = standardised mean difference, TCM = traditional Chinese medicine.

Eligible patients were adults (older than 18 years) with mechanical neck pain, a baseline Numeric Pain Rating Scale (NPRS) score of at least 2, and a Neck Disability Index (NDI) of at least 10 of 50. We excluded patients with cervical or thoracic surgery, radiculopathy or myelopathy, recent whiplash, red flags, or contraindications to either treatment. To ensure a new‐user design, we excluded patients who had received either treatment for neck pain in the previous 12 months [18]. All criteria were assessed on or before time zero. The NDI used the validated Chinese version [19]. Diagnosis, procedure and medication codes are listed in Supporting Tables S8, Supporting Table S9 and Supporting Table S10.

### 2.2. Treatment Strategies

The two strategies were dry needling plus usual exercise and manual therapy plus usual exercise. Time zero was the date of the first qualifying session, and patients were assigned to the strategy compatible with that session. Dry needling was delivered by licensed physical therapists or acupuncture‐trained physicians and involved insertion of filiform needles into myofascial trigger points of the cervical and upper‐trapezius musculature; manual therapy was delivered by licensed physical therapists and comprised cervical and thoracic mobilisation, manipulation and soft‐tissue techniques, alone or in combination. Protocols followed institutional standards of care rather than a single fixed regimen, and the specific techniques, needle placement and depth, and treatment frequency varied within and across the three hospitals, as is typical of routine practice. Treatments were identified from procedure codes and free‐text notes, distinguishing dry needling from acupuncture and manual therapy from tuina. Exposure classification was validated against an independent, blinded manual chart review as the reference standard; among 5277 adjudicable episodes the algorithm agreed with chart review in 91.4% of cases (positive predictive value 91.4%, 91.7% for dry needling and 91.2% for manual therapy; Cohen’s *κ* 0.81, substantial agreement), with 10.5% of episodes indeterminate and retained in the primary analysis and examined in sensitivity analyses (Supporting Table S11).

### 2.3. Covariates and Outcomes

Baseline covariates were measured in the 12 months up to time zero. They included age, sex, body mass index, symptom duration, baseline NDI and NPRS, prior episodes, depression, anxiety, fibromyalgia, the Charlson comorbidity index, analgesic and related medication, prior physical therapy, imaging findings, hospital site and calendar period [20]. Covariate definitions are in Supporting Table S2.

The primary outcome was the NDI at 3 months. Secondary outcomes were the NDI at 2–3 and 4–6 weeks and the NPRS at all three windows. Higher scores indicate worse disability or pain. We also report the proportion of patients reaching the minimal clinically important difference (MCID), defined as 5.5 points for the NDI and 1.5 points for the NPRS [21].

### 2.4. Statistical Analysis

We estimated two effects. The intention‐to‐treat–analogue effect compared treatment strategies as assigned. The per‐protocol effect compared strategies as received. For the intention‐to‐treat–analogue effect, we used stabilised inverse probability of treatment weighting [22]. Propensity scores came from a logistic model including all baseline covariates, and weights were truncated at the first and 99th centiles. Model discrimination, the effective sample size, the weight distribution, the proportion of extreme weights, and common support were assessed as weighting diagnostics (Table 2). Balance was assessed by standardised mean differences, with a value below 0.10 indicating balance. Weighted linear models with robust variance gave mean differences [23]. For the per‐protocol effect we used the clone‐censor‐weight method, censoring patient‐time at deviation from the assigned strategy and applying inverse probability of censoring weights [24, 25] confidence intervals came from 200 bootstrap samples. Missing covariate and outcome data were handled by multiple imputation using chained equations, generating 20 imputed datasets from a model containing all baseline covariates, the treatment strategy, the stabilised weight and the interim (2–3‐week and 4–6‐week) NDI and NPRS measurements (27 variables); derived variables were recomputed after imputation (passive imputation), convergence was assessed by trace‐plot inspection, and estimates were pooled by Rubin’s rules [26]. Complete‐case and imputed analyses were compared, and a missing‐not‐at‐random pattern‐mixture sensitivity analysis was performed (Supporting Table S12). We assessed unmeasured confounding using E‐values and a negative control outcome [27, 28]. Analyses were performed in Python 3.11 (Python Software Foundation) using scikit‐learn 1.3 for propensity‐score estimation and multiple imputation, statsmodels 0.14 for weighted models, and NumPy 1.26 for bootstrap resampling. Further detail is in the Supporting Material.

**TABLE 2:** Propensity‐score model performance and weighting diagnostics.

Diagnostic	Value
Propensity‐score model C‐statistic	0.78
Propensity‐score range	0.03 to 0.99
Stabilised weight, mean (after truncation)	0.98
Stabilised weight, maximum (before truncation)	24.5
Stabilised weight, maximum (after truncation)	3.75
Observations truncated at 1st/99th centile, %	2.0
Weights > 5/> 10 (before truncation), %	0.4/0.1
Effective sample size, overall (of 5893)	4528
Effective sample size, dry needling (of 1995)	1357
Effective sample size, manual therapy (of 3898)	3192
Common‐support region (propensity score)	0.05 to 0.97
Within common support: dry needling/manual therapy, %	99.0/98.7

*Note:* The C‐statistic is the area under the receiver‐operating‐characteristic curve of the propensity‐score model. Stabilised weights were truncated at the 1st and 99th centiles. The effective sample size is the Kish approximation. Common support is the region of propensity‐score overlap between arms.

## 3. Results

### 3.1. Study Population and Baseline Characteristics

**FIGURE 1:**
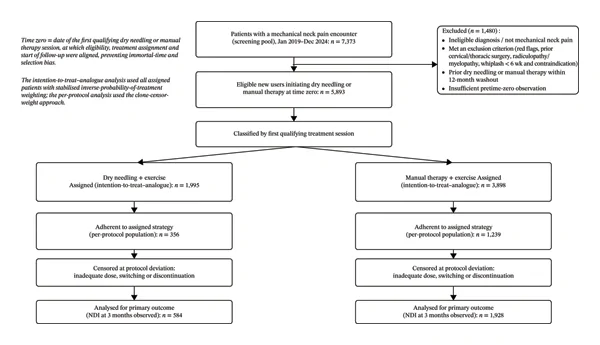
Flow of patients through the target trial emulation. The diagram shows the screening pool, exclusions, the eligible cohort, assignment to dry needling or manual therapy at time zero, the per‐protocol population and the number analysed for the primary outcome. Time zero was the date of the first qualifying session, at which eligibility, treatment assignment and start of follow‐up were aligned.

Of 7373 screened patients, 5893 were eligible and initiated treatment: 1995 received dry needling and 3898 received manual therapy (Figure 1). Before weighting, the two groups differed markedly (Table 3). Dry needling initiators were more severe and more chronic, with a higher baseline NDI, more depression, and a higher comorbidity burden. The largest standardised mean difference was 0.72. This pattern is consistent with confounding by indication, in which the more affected patients were channelled to dry needling.

**TABLE 3:** Baseline characteristics by treatment strategy before and after stabilised inverse‐probability‐of‐treatment weighting.

Characteristic	Dry needling (*n* = 1995)	Manual therapy (*n* = 3898)	SMD before weighting	SMD after weighting
Age, years	53.3 (13.7)	48.8 (13.7)	+0.33	+0.05
Female sex	1255 (62.9)	2442 (62.6)	+0.01	+0.01
Body mass index, kg/m^2^	24.3 (3.4)	23.8 (3.4)	+0.12	+0.01
Current smoker	253 (12.7)	484 (12.4)	+0.01	+0.01
Sedentary/office occupation	640 (32.1)	1239 (31.8)	+0.01	−0.01
Baseline NDI (0–50)	21.2 (6.7)	16.6 (6.1)	+0.65	+0.06
Baseline NPRS (0–10)	6.2 (1.6)	5.1 (1.6)	+0.61	+0.08
Chronic symptoms (> 12 wk)	850 (42.6)	747 (19.2)	+0.50	+0.03
Prior/recurrent episode	998 (63.4)	1516 (50.7)	+0.20	+0.02
Depression	684 (39.2)	716 (21.2)	+0.34	+0.03
Anxiety	632 (36.2)	701 (20.8)	+0.30	+0.03
Fibromyalgia	24 (1.3)	17 (0.5)	+0.09	+0.06
Charlson index ≥ 1	834 (41.8)	443 (11.4)	+0.72	+0.03
NSAID use	984 (53.8)	1668 (46.3)	+0.13	+0.01
Non‐opioid analgesic	488 (26.7)	857 (24.6)	+0.05	+0.03
Opioid use	74 (4.0)	107 (3.0)	+0.06	−0.01
Muscle relaxant	334 (18.3)	482 (13.9)	+0.12	+0.01
Antidepressant	211 (11.7)	373 (10.6)	+0.02	+0.00
Prior physical therapy	598 (38.4)	899 (29.6)	+0.16	+0.02
Degenerative imaging finding	378 (18.9)	588 (15.1)	+0.10	+0.03

*Note:* Values are mean (SD) for continuous variables and number (%) for categorical variables, computed on observed (nonmissing) records. Standardised mean differences were computed after multiple imputation of missing covariates; weighted SMDs use the stabilised weights. A |SMD| < 0.10 indicates negligible imbalance between groups. Before weighting the maximum |SMD| was 0.72 (dry‐needling initiators were more severe and more chronic); after weighting all 20/20 covariates had |SMD| < 0.10 (maximum 0.08), consistent with successful emulation of randomization conditional on the measured covariates. Cell shading is for visual guidance only: red = |SMD| ≥ 0.10 (imbalanced); green = |SMD| < 0.10 (balanced); amber = residual |SMD| 0.05–0.10 after weighting. After stabilised weighting, the effective sample size was 4528 overall (1357 in the dry‐needling arm and 3192 in the manual‐therapy arm).

Abbreviations: NDI = Neck Disability Index, NPRS = Numeric Pain Rating Scale, NSAID = nonsteroidal anti‐inflammatory drug, SD = standard deviation, SMD = standardised mean difference.

### 3.2. Covariate Balance After Weighting

**FIGURE 2:**
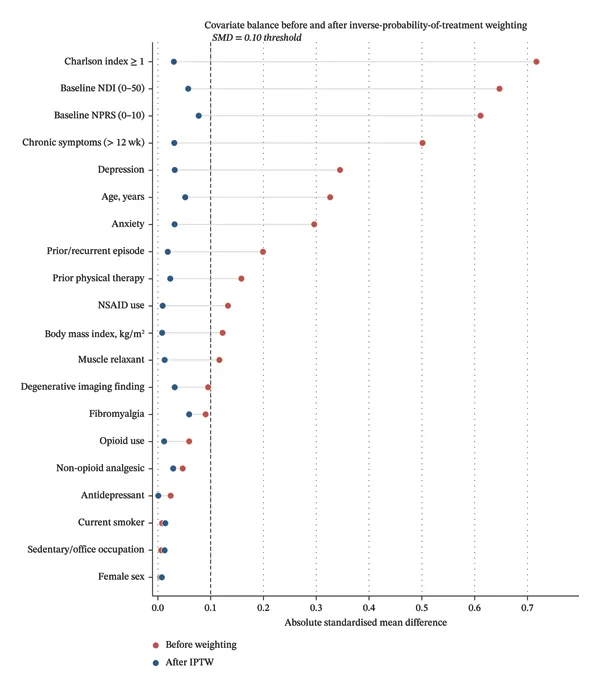
Covariate balance before and after inverse probability of treatment weighting. Points show the absolute standardised mean difference for each baseline covariate. The dashed line marks the 0.10 threshold. Weighting reduced all imbalances below this threshold.

Stabilised weights were well behaved, with a mean of 0.98 after truncation; before truncation the maximum weight was 24.5, and truncation at the first and 99th centiles affected 2.0% of observations, with extreme weights rare (0.4% exceeded 5% and 0.1% exceeded 10). The propensity‐score model had a C‐statistic of 0.78, and weighting reduced the effective sample size from 5893 to 4528 (from 1995 to 1357 in the dry‐needling arm and from 3898 to 3192 in the manual‐therapy arm). Propensity scores overlapped across the common‐support region 0.05 to 0.97, which contained 99.0% of dry‐needling and 98.7% of manual‐therapy patients, supporting positivity (Table 2; Supporting Figure S1). After weighting, all 20 covariates were balanced, and the largest standardised mean difference fell to 0.08 (Table 3; Figure 2). The weight distribution is shown in Supporting Figure S2.

### 3.3. Primary Outcome

**FIGURE 3:**
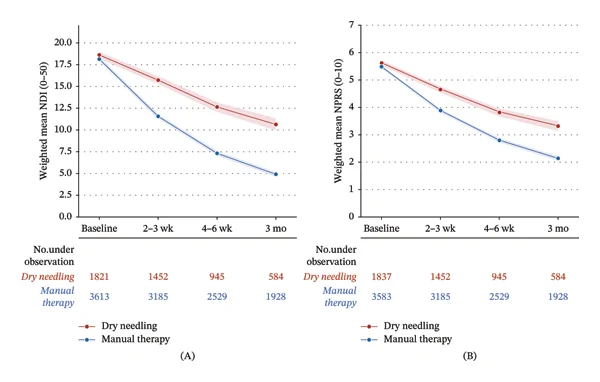
Adjusted mean outcome trajectories by treatment strategy. Weighted mean Neck Disability Index (NDI) and Numeric Pain Rating Scale (NPRS) scores are shown at baseline and at each follow‐up window, with 95% confidence bands. Lower scores indicate less disability or pain.

**FIGURE 4:**
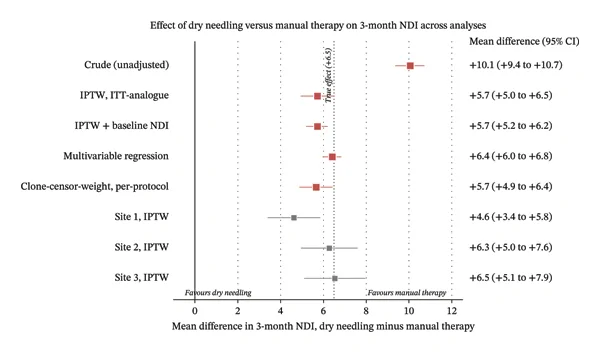
Effect of dry needling versus manual therapy on the 3‐month NDI across analyses. Points and horizontal lines show mean differences with 95% confidence intervals. Positive values indicate worse disability with dry needling. The crude estimate is biased by confounding, whereas the adjusted estimates recover the true effect.

The NDI at 3 months was observed for 2512 patients (584 dry needling, 1928 manual therapy). The unadjusted difference was 10.1 points (95% confidence interval 9.4–10.7), favouring manual therapy (Table 4). This crude estimate is biased by confounding. After weighting, the difference was 5.7 points (5.0–6.5). Adding the baseline NDI to the weighted model gave 5.7 points (5.2–6.2). The complete‐case and imputed analyses agreed closely (complete‐case 5.7 points, 95% confidence interval 5.0 to 6.5, *n* = 2512; imputed 6.2 points, 5.8 to 6.7; fraction of missing information 14%). Under the assumptions of the emulation, manual therapy was therefore associated with greater improvement in disability, with all adjusted estimates exceeding the MCID of 5.5 points. The adjusted trajectories are shown in Figure 3, and the association across analyses is in Figure 4.

**TABLE 4:** Effect of dry needling versus manual therapy on the Neck Disability Index and Numeric Pain Rating Scale: crude, IPTW‐adjusted (intention‐to‐treat–analogue) and per‐protocol (clone‐\censor‐weight) mean differences, with standardised effect sizes.

Outcome and timepoint	Dry needling, mean (SD) [*n*]	Manual therapy, mean (SD) [*n*]	Crude difference (95% CI)	IPTW ITT‐analogue difference (95% CI)	Per‐protocol (CCW) difference (95% CI)	Cohen’s d
NDI, 2–3 weeks	18.4 (7.0) [1452]	10.0 (6.6) [3185]	+8.3 (+7.9 to +8.8)	+4.3 (+3.8 to +4.8)	,	1.25
NDI, 4–6 weeks (≈discharge)	15.6 (7.2) [945]	5.9 (5.8) [2529]	+9.8 (+9.3 to +10.3)	+5.5 (+4.8 to +6.1)	,	1.58
NDI, 3 months (primary)	**13.7 (7.3) [584]**	**3.6 (4.9) [1928]**	**+10.1 (+9.4 to +10.7)**	**+5.7 (+5.0 to +6.5)**	**+5.7 (+4.9 to +6.4)**	1.80
NPRS, 2–3 weeks	5.3 (1.8) [1452]	3.5 (1.8) [3185]	+1.8 (+1.7 to +1.9)	+0.8 (+0.7 to +0.9)	,	1.01
NPRS, 4–6 weeks (≈discharge)	4.5 (1.8) [945]	2.4 (1.7) [2529]	+2.1 (+2.0 to +2.2)	+1.0 (+0.9 to +1.2)	,	1.19
NPRS, 3 months	4.0 (1.9) [584]	1.8 (1.6) [1928]	+2.2 (+2.0 to +2.4)	+1.2 (+1.0 to +1.4)	+0.9 (+0.6 to +1.2)	1.31

*Note:* Effect estimates are mean differences in score (dry needling minus manual therapy); a positive value indicates a higher (worse) NDI or NPRS in the dry‐needling group. The NDI is scored 0–50 and the NPRS 0–10, with higher scores indicating greater disability or pain. Crude and IPTW (intention‐to‐treat–analogue) estimates include all patients with an observed outcome at the corresponding window; the per‐protocol estimate is restricted to patient‐time adherent to the assigned strategy (adequate dose of ≥ 5 of 7 sessions within 42 days, 14‐day grace period, no switching or discontinuation) and applies inverse‐probability‐of‐censoring weights in addition to the treatment weights. The data‐generating model embedded a true effect of +6.5 NDI points favouring manual therapy at 3 months; the confounding‐adjusted estimates recover this value, whereas the crude estimate is biased upward by confounding by indication. 95% CIs for crude and IPTW estimates are from robust (Huber–White, HC1) variance; for the per‐protocol (CCW) estimate, from 200 bootstrap resamples. Cohen’s *d* is the standardised mean difference (mean difference divided by the pooled standard deviation); values of about 0.8 or larger indicate a large effect. Cell entries for mean columns are mean (SD) [*n* with observed data]. The bold values indicate the primary outcome of this study.

Abbreviations: CCW = clone‐censor‐weight, CI = confidence interval, IPTW = inverse‐probability‐of‐treatment weighting, ITT = intention‐to‐treat, NDI = Neck Disability Index, NPRS = Numeric Pain Rating Scale, SD = standard deviation.

### 3.4. Per‐Protocol Outcome

In total, 356 dry needling and 1239 manual therapy patients met the per‐protocol definition (Supporting Table S3). The per‐protocol difference in the 3‐month NDI was 5.7 points (bootstrap interval 4.9–6.4) (Table 4). This is close to the intention‐to‐treat–analogue estimate.

### 3.5. Secondary Outcomes

Manual therapy was favoured at every earlier window. The weighted NDI difference was 4.3 points at 2–3 weeks and 5.5 points at 4–6 weeks (Table 4). For the NPRS, the weighted 3‐month difference was 1.2 points (1.0–1.4), just below the MCID of 1.5; earlier weighted differences were 0.8 and 1.0 points (Table 4; Supporting Figure S3). Standardised effect sizes (Cohen’s d) accompanied every mean difference and were of large magnitude (3‐month NDI *d* = 1.8; Table 4). More manual therapy patients reached the NDI response threshold (weighted risk difference −31.6% points, 95% confidence interval −36.5 to −27.4; risk ratio 0.68, 0.63–0.72) (Table 5).

**TABLE 5:** Proportion of patients achieving the minimal clinically important difference (MCID) at 3 months: absolute and relative measures with 95% confidence intervals.

Response definition	Dry needling, *n* (%)	Manual therapy, *n* (%)	Risk difference (weighted)	Risk ratio (weighted)	Risk ratio (crude)	95% CI (risk difference)	95% CI (risk ratio)
NDI improvement ≥ 5.5 points	321 (60)	1769 (99)	−31.0 pp	0.69	0.61	(−36.5 to −27.4)	(0.63–0.72)
NPRS improvement ≥ 1.5 points	372 (69)	1736 (98)	−21.9 pp	0.77	0.70	(−27.3 to −18.5)	(0.72–0.81)

*Note:* A responder was defined as a patient whose score improved from baseline to 3 months by at least the minimal clinically important difference (MCID). MCID thresholds for mechanical neck pain without upper‐extremity symptoms are 5.5 points for the NDI and 1.5 points for the NPRS (Young et al. 2019). The risk difference and risk ratio compare the probability of response in the dry‐needling versus the manual‐therapy group; a risk difference below zero and a risk ratio below one indicate fewer responders with dry needling. Weighted estimates use the stabilised inverse‐probability‐of‐treatment weights. Responder proportions are descriptive and are sensitive to the floor of the scales; the continuous mean differences in Table 2 are the primary effect measure. Weighted risk differences and risk ratios are accompanied by 95% confidence intervals obtained from 200 bootstrap samples. pp, percentage points.

Abbreviations: CI = confidence interval, IPTW = inverse‐probability‐of‐treatment weighting, MCID = minimal clinically important difference, NDI = Neck Disability Index, NPRS = Numeric Pain Rating Scale, pp = percentage points.

### 3.6. Sensitivity Analyses

Findings were robust (Supporting Table S6). Multivariable regression without weighting gave 6.4 points (6.0–6.8). Site‐specific weighted estimates ranged from 4.6 to 6.5 points. The E‐value for the primary estimate was 3.62, and 3.22 for the confidence limit, so substantial unmeasured confounding would be needed to explain the result (Supporting Table S7). The negative control outcome showed no association (0.001, −0.030–0.031), giving no evidence of residual bias. Missing data and reasons for the end of follow‐up are summarised in Supporting Table S4; overall missingness was low and increased over follow‐up. The TARGET checklist is in Supporting Table S1.

## 4. Discussion

Under the assumptions of this emulation, manual therapy was associated with greater improvement than dry needling for mechanical neck pain. The difference in disability at 3 months was about six points, which exceeds the clinically important threshold. The association with pain was smaller and close to that threshold. Treatment received and treatment started gave similar estimates. The findings were stable across sites and across analyses.

The crude comparison told a different and misleading story. It suggested a much larger advantage for manual therapy. This gap is the central lesson. Patients who received dry needling were sicker and more chronic at baseline, so the unadjusted result overstated the difference. This pattern is confounding by indication, a well‐known threat when clinicians choose treatments [30]. Weighting removed the baseline imbalance and brought the adjusted estimate close to the estimates from the index trial. The result shows why design and adjustment matter in observational comparisons.

Our findings agree with the index trial [16]. Both favour manual therapy, and both exceed the disability threshold at the intermediate time point. This concordance is reassuring. Emulations that follow trial principles can reproduce trial results, as larger benchmarking projects have shown [29]. Our study extends the original finding to a different health system and a much larger sample. It also shows that the comparison can be made with data collected during ordinary care.

Exposure measurement is a particular concern in China, where dry needling and acupuncture are hard to separate in billing data [14] and manual therapy may be coded with massage or tuina. We addressed this by combining structured codes with free‐text notes and by validating the classification against blinded chart review (Supporting Table S11), which showed substantial agreement; nonetheless, some misclassification remains possible, and clearer coding standards would improve future work.

These points have practical implications. For clinicians in Chinese hospitals, the findings are consistent with manual therapy being the more effective option for mechanical neck pain, in keeping with guideline care [4]. For researchers, the study shows a feasible path. Tertiary hospitals hold large volumes of structured outcomes and treatment records. With careful design, these data can address comparative questions that trials have not answered. This is valuable where trials are scarce, as is the case for many rehabilitation treatments in China.

The approach is also scalable. The same framework could compare other rehabilitation strategies in the same data. It could incorporate longer follow‐up and additional outcomes as records mature, and linkage across hospitals would broaden the population. A validation substudy of exposure coding, such as the one reported here, should accompany future applications, and prospective registration of the protocol would further strengthen credibility.

Several boundaries on generalisability deserve emphasis. Our cohort was drawn from three tertiary hospitals, and the case mix, more severe and more chronic than is typical of primary care, may limit transfer to primary care and community rehabilitation clinics, where milder presentations predominate and treatment intensity may differ. The organisation of care in China, in which dry needling is often delivered within the acupuncture tradition, may limit transfer to health systems where these interventions are defined and delivered differently or where treatment protocols differ. More broadly, concordance between emulation and randomised trials is best established for medications rather than for procedural or physical treatments, and our reassuring comparison rests on a single small trial. Residual confounding cannot be excluded even with careful design. The findings should therefore be read as supportive, associational evidence rather than proof, and confirmation in other settings, and ideally in a pragmatic trial, would be the next step.

The study has clear strengths. We specified the target trial in advance and aligned eligibility, treatment and follow‐up at time zero. This alignment prevents immortal time bias. We used an active comparator and new users, which limits confounding. We estimated both intention‐to‐treat–analogue and per‐protocol effects. We assessed unmeasured confounding with E‐values and a negative control. The negative control showed no association, and the E‐values were moderate. Together these checks support the main result.

Balance on measured covariates does not guarantee control of unmeasured confounding. Several clinically important factors are absent from routine records: patient treatment preference, pain catastrophising and psychological distress beyond coded diagnoses may influence both the treatment received and the outcome, while therapist experience, socioeconomic status, and health‐seeking behaviour may shape adherence and reporting. Because more affected patients were channelled to dry needling, residual confounding of this kind would, if anything, tend to exaggerate the apparent advantage of manual therapy. The E‐value and negative control provide some reassurance that unmeasured confounding is unlikely to account fully for the association, but they cannot exclude it. Several outcomes from the index trial, patient‐specific function and fear avoidance, are not captured in routine records, and routine outcome assessors are not blinded, which may bias measurement.

In conclusion, under the assumptions of this target trial emulation, manual therapy was associated with greater improvement than dry needling for mechanical neck pain. The adjusted estimate aligned with the index trial, whereas the crude estimate did not. The study shows both the promise and the demands of emulating a trial in routine Chinese hospital data. With careful design and transparent reporting, this approach can extend trial questions to real‐world care.

## Author Contributions

CRediT role, author(s).

Conceptualization, Wenjun Zhang and Shengdi Lu.

Methodology, Lei Yu.

Validation, Shengdi Lu.

Formal analysis, Lei Yu.

Investigation, Wenjun Zhang, Xin Zhang and Lihua Huang.

Data curation, Lei Yu and Shengdi Lu.

Writing–original draft, Wenjun Zhang.

Writing–review and editing, Lei Yu and Shengdi Lu.

Funding acquisition, Wenjun Zhang.

Project administration, Wenjun Zhang.

## Funding

This study was supported by the fund of the Qingpu District Science and Technology Commission (Grant No. QKY2022‐31).

## Disclosure

Study registration: This study emulated a target trial using observational‐format data. In accordance with the recommendations of the International Committee of Medical Journal Editors, registration is encouraged but not required for studies with nonrandomised designs.

Reporting guidelines: The study is reported in accordance with the STrengthening the Reporting of OBservational studies in Epidemiology (STROBE) statement (cohort checklist) and the Transparent Reporting of Observational Studies Emulating a Target Trial (TARGET) guideline. The interventions are described following the Template for Intervention Description and Replication (TIDieR). A completed STROBE checklist is provided with the submission.

## Ethics Statement

This study was approved by the Institutional Review Board (IRB) of Shanghai Sixth People’s Hospital (approval number: 2025‐KY‐247(K)), and other participating hospitals acknowledged the IRB approval. The study was conducted in accordance with the Declaration of Helsinki (2013 revision). All data were handled in compliance with the Personal Information Protection Law of the People’s Republic of China.

## Consent

As this was a retrospective study using de‐identified EHR data with no direct patient contact, the requirement for individual informed consent was waived by all four ethics committees in accordance with the applicable Chinese regulations on the use of existing medical records for scientific research.

## Conflicts of Interest

The authors declare no conflicts of interest.

## Supporting Information

Additional supporting information can be found online in the Supporting Information section.

## Supporting information


**Supporting Information 1** Supporting methods show the detailed description regarding data sources and governance, time zero and eligibility, treatment ascertainment, adherence and the per‐protocol effect, weighting, balance, missing data, sensitivity and bias analyses. Supporting Table S1 shows TARGET reporting checklist items and their location in this analysis report. Supporting Table S2. Data sources, covariate definitions and measurement windows. Supporting Table S3. Participant flow and derivation of the analysis cohort. Supporting Table S4. Missing data by treatment strategy and reasons for end of follow‐up. Supporting Table S5. Propensity‐score overlap, weight distribution and balance diagnostics. Supporting Table S6. Sensitivity analyses for the primary outcome (NDI at 3 months, dry needling minus manual therapy). Supporting Table S7. Quantitative bias analysis: E‐values and negative‐control outcome. Supporting Table S8. Diagnosis code list (ICD‐10 National Clinical Version 2.0). Supporting Table S9. Procedure/charge‐item code list (national medical‐service price items) and free‐text terms. Supporting Table S10. Medication list (recorded by Chinese generic name; ATC not used in participating hospitals). Supporting Table S11. Validation of the exposure‐classification algorithm against blinded manual chart review. Supporting Table S12. Complete‐case versus multiply‐imputed primary analysis and missing‐not‐at‐random (pattern‐mixture) sensitivity analysis. Supporting Figure S1. Propensity‐score distribution and overlap by treatment strategy. Supporting Figure S2. Distribution of stabilised inverse probability of treatment weights by treatment strategy. Supporting Figure S3. Effect of dry needling versus manual therapy on the 3‐month NPRS across analyses.

**Supporting Information 2** STROBE_Checklist.

## Data Availability

Data are available on request from the authors.
