# Supplementary material for: Dry Needling Versus Manual Therapy for Mechanical Neck Pain: A Target Trial Emulation Using Electronic Health Records
Source: Pain Res Manag. 2026 Aug 28;2026:3221855. doi: 10.1155/prm/3221855 (PMC13522986; doi:10.1155/prm/3221855)
Supplement: Supplementary file 1 [file PRM-2026-3221855-s001.docx]

**STROBE Statement — Checklist of items that should be included in reports of cohort studies**

*Manuscript: Dry needling versus manual therapy for mechanical neck pain: a target trial emulation using electronic health records.*

| **Item** | **Recommendation** | **Location in manuscript / Supplementary Material** | **Reported** |
| --- | --- | --- | --- |
| **Title and abstract** | | | |
| **1 (a)** | Indicate the study’s design with a commonly used term in the title or the abstract. | Title; Abstract (Design, Setting, and Participants) — identified as a “target trial emulation”. | Yes |
| **1 (b)** | Provide in the abstract an informative and balanced summary of what was done and what was found. | Abstract (structured: Importance, Objective, Design, Interventions, Outcomes, Results, Conclusions). | Yes |
| **Introduction** | | | |
| **2** | Explain the scientific background and rationale for the investigation being reported. | Background, paragraphs 1–5. | Yes |
| **3** | State specific objectives, including any prespecified hypotheses. | Background, final paragraph (aim, objective, and hypothesis). | Yes |
| **Methods** | | | |
| **4** | Present key elements of study design early in the paper. | Methods — Study design; Table 1 (target trial specification and emulation). | Yes |
| **5** | Describe the setting, locations, and relevant dates, including periods of recruitment, exposure, follow-up, and data collection. | Methods — Study design (three tertiary hospitals in China, January 2019 to December 2024); Supplementary methods S1. | Yes |
| **6 (a)** | Cohort study—Give the eligibility criteria, and the sources and methods of selection of participants. Describe methods of follow-up. | Methods — Study population; Supplementary methods S2; Supplementary Table S3 (flow). | Yes |
| **6 (b)** | Cohort study—For matched studies, give matching criteria and number of exposed and unexposed. | Not applicable: groups were balanced by inverse probability of treatment weighting rather than matching (Methods — Statistical analysis). | Yes |
| **7** | Clearly define all outcomes, exposures, predictors, potential confounders, and effect modifiers. Give diagnostic criteria, if applicable. | Methods — Treatments under comparison, Covariates, Outcomes; Supplementary Table S2; Supplementary Tables S8–S10 (code lists). | Yes |
| **8** | For each variable of interest, give sources of data and details of methods of assessment (measurement). Describe comparability of assessment methods if there is more than one group. | Methods — Treatments under comparison, Outcomes; Supplementary methods S1, S3; Supplementary Table S2. | Yes |
| **9** | Describe any efforts to address potential sources of bias. | Methods — Study design (time zero alignment), Statistical analysis; Supplementary methods S2–S6; sensitivity and bias analyses. | Yes |
| **10** | Explain how the study size was arrived at. | Methods — Study population; Supplementary Table S3 (derivation of the analysis cohort from the screening pool). | Yes |
| **11** | Explain how quantitative variables were handled in the analyses. If applicable, describe which groupings were chosen and why. | Methods — Statistical analysis, Outcomes (continuous NDI and NPRS; minimal clinically important difference thresholds). | Yes |
| **12 (a)** | Describe all statistical methods, including those used to control for confounding. | Methods — Statistical analysis (IPTW, clone-censor-weight); Supplementary methods S4–S6. | Yes |
| **12 (b)** | Describe any methods used to examine subgroups and interactions. | Sensitivity analyses (site-stratified estimates); Supplementary Table S6. | Yes |
| **12 (c)** | Explain how missing data were addressed. | Methods — Statistical analysis (multiple imputation by chained equations); Supplementary methods S5; Supplementary Table S4. | Yes |
| **12 (d)** | Cohort study—If applicable, explain how loss to follow-up was addressed. | Sensitivity analyses; Supplementary Table S4 (reasons for end of follow-up, including loss to follow-up). | Yes |
| **12 (e)** | Describe any sensitivity analyses. | Results — Sensitivity analyses; Supplementary methods S6; Supplementary Tables S6–S7. | Yes |
| **Results** | | | |
| **13 (a)** | Report the numbers of individuals at each stage of the study—numbers potentially eligible, examined for eligibility, confirmed eligible, included in the study, completing follow-up, and analysed. | Results — Study population and baseline characteristics; Figure 1; Supplementary Table S3. | Yes |
| **13 (b)** | Give reasons for non-participation at each stage. | Figure 1; Supplementary Table S3 (exclusions); Supplementary Table S4 (end-of-follow-up reasons). | Yes |
| **13 (c)** | Consider use of a flow diagram. | Figure 1 (flow of patients through the emulation). | Yes |
| **14 (a)** | Give characteristics of study participants and information on exposures and potential confounders. | Results — Study population and baseline characteristics; Table 2. | Yes |
| **14 (b)** | Indicate the number of participants with missing data for each variable of interest. | Supplementary Table S4 (missing data by treatment strategy). | Yes |
| **14 (c)** | Cohort study—Summarise follow-up time (eg, average and total amount). | Methods — Outcomes (follow-up windows at 2–3 weeks, 4–6 weeks, and 3 months); Supplementary Table S4. | Yes |
| **15** | Cohort study—Report numbers of outcome events or summary measures over time. | Results — Primary and Secondary outcomes; Table 3; Figure 3. | Yes |
| **16 (a)** | Give unadjusted estimates and, if applicable, confounder-adjusted estimates and their precision (eg, 95% confidence interval). Make clear which confounders were adjusted for and why they were included. | Results — Primary outcome; Table 3 (crude, IPTW, and per-protocol estimates with 95% CIs); Figure 4. | Yes |
| **16 (b)** | Report category boundaries when continuous variables were categorized. | Table 4 (minimal clinically important difference thresholds for responder definitions: NDI ≥5.5; NPRS ≥1.5). | Yes |
| **16 (c)** | If relevant, consider translating estimates of relative risk into absolute risk for a meaningful time period. | Table 4 (absolute risk differences and risk ratios for achieving the minimal clinically important difference at 3 months). | Yes |
| **17** | Report other analyses done—eg analyses of subgroups and interactions, and sensitivity analyses. | Results — Sensitivity analyses; Supplementary Tables S6–S7 (site-stratified estimates, E-values, negative control). | Yes |
| **Discussion** | | | |
| **18** | Summarise key results with reference to study objectives. | Discussion, paragraphs 1–3. | Yes |
| **19** | Discuss limitations of the study, taking into account sources of potential bias or imprecision. Discuss both direction and magnitude of any potential bias. | Discussion, paragraphs 5–6 and the caution paragraph; Supplementary methods S7. | Yes |
| **20** | Give a cautious overall interpretation of results considering objectives, limitations, multiplicity of analyses, results from similar studies, and other relevant evidence. | Discussion, paragraphs 3–4 and 7–10 (interpretation, comparison with the index trial, implications, and caution). | Yes |
| **21** | Discuss the generalisability (external validity) of the study results. | Discussion, paragraphs 3 and 7–9 (extension to a different health system; implications for Chinese hospitals). | Yes |
| **Other information** | | | |
| **22** | Give the source of funding and the role of the funders for the present study and, if applicable, for the original study on which the present article is based. | Statements and Declarations — Role of the funding source (also a subsection of Methods). | Yes |
