# Supplementary material for: Dry Needling Versus Manual Therapy for Mechanical Neck Pain: A Target Trial Emulation Using Electronic Health Records
Source: Pain Res Manag. 2026 Aug 28;2026:3221855. doi: 10.1155/prm/3221855 (PMC13522986; doi:10.1155/prm/3221855)
Supplement: Supplementary file 2 [file PRM-2026-3221855-s002.docx]

**Supplementary Material**

**Supplementary methods**

**S1. Data sources and governance**

Data came from the EHRs of three tertiary (Class A) hospitals in China for January 2019 to December 2024. Records included structured demographics, diagnoses, procedure and charge items, medication orders, rehabilitation outcome tables, imaging reports, and free-text notes. Diagnoses used codes from the International Classification of Diseases, 10th Revision (China National Clinical Version 2.0). Medications were recorded by Chinese generic name; the Anatomical Therapeutic Chemical system is not used in these hospitals. Site was retained as a stratification variable.

**S2. Time zero and eligibility**

Person-time was divided into encounter-anchored intervals, and eligibility was assessed at each candidate initiation. Time zero was the date of the first qualifying dry needling or manual therapy session. Eligibility, treatment assignment, and start of follow-up were aligned at this date. All eligibility variables were drawn from on or before time zero, with condition-specific lookbacks for exclusions. The primary analysis used the first eligible initiation per patient. A sequential, encounter-anchored emulation was prespecified as a sensitivity analysis, with robust variance for repeated contributions.

**S3. Treatment ascertainment**

Dry needling and manual therapy were identified from both structured procedure or charge items and free-text notes. Dry needling may be billed under acupuncture items, and manual therapy under tuina or massage. A rule-based and machine-learning pipeline classified each session, and discordant records were adjudicated by an independent, blinded manual chart review that served as the reference standard. Agreement between the algorithm and chart review was quantified by the positive predictive value and Cohen’s κ (Supplementary Table S11). Among the 5277 episodes that could be adjudicated, the algorithm agreed with chart review in 91.4% of cases (positive predictive value 91.4% overall; 91.7% for dry needling and 91.2% for manual therapy; κ 0.81). A further 616 episodes (10.5%) were indeterminate on review; these were retained in the primary analysis and were removed in a sensitivity analysis, which did not materially change the results. Because the reference standard was applied to episodes already selected as dry needling or manual therapy, sensitivity and specificity for a single exposure against the full source population are not identifiable, and the positive predictive value and κ are the appropriate validation metrics for this design.

**S4. Adherence and the per-protocol effect**

Mirroring the index trial dose of seven sessions over six weeks, adequate dose was defined as at least five sessions within 42 days. Protocol deviation was defined as inadequate dose, switching to the comparator, or discontinuation before adequate dose, with a 14-day grace period. For the per-protocol effect we used the clone-censor-weight method. Patients were assigned to the strategy compatible with their initiation, patient-time was censored at deviation, and inverse probability of censoring weights were applied. These weights were combined with the treatment weights, and confidence intervals came from 200 bootstrap samples.

**S5. Weighting, balance, and missing data**

Propensity scores for dry needling were estimated by logistic regression on all baseline covariates. Stabilized weights were truncated at the first and 99th centiles. Balance was summarized by standardized mean differences, with values below 0.10 indicating balance. Positivity was checked by inspecting propensity-score overlap and the common-support region. Missing covariate and outcome data were handled by multiple imputation using chained equations, generating 20 imputed datasets. The imputation model included all baseline covariates, the treatment strategy, the stabilized weight, and the interim (2–3-week and 4–6-week) Neck Disability Index and Numeric Pain Rating Scale measurements, giving 27 variables in total; the inclusion of the strongly predictive interim outcomes supports the plausibility of the missing-at-random assumption. Continuous variables were imputed by predictive mean matching within the chained-equations algorithm, and any variable that was a deterministic function of others was recomputed after imputation (passive imputation) rather than imputed directly. Convergence was assessed by inspecting trace plots of the imputed means and variances across iterations, which showed no trend and adequate mixing. Estimates were pooled across the 20 completed datasets using Rubin’s rules. Outcome missingness increased over follow-up and was higher in the dry-needling group and in more severe patients; the complete-case and imputed analyses agreed closely, and a missing-not-at-random pattern-mixture sensitivity analysis (Supplementary Table S12) confirmed that the direction and significance of the association were preserved.

**S6. Sensitivity and bias analyses**

We prespecified several sensitivity analyses: a baseline-adjusted weighted model; multivariable outcome regression without weighting; site-stratified weighted estimates; the clone-censor-weight per-protocol analysis; E-values for unmeasured confounding; and a negative control outcome with no plausible link to treatment. The continuous mean difference for the primary outcome was converted to an approximate risk-ratio scale before computing the E-value.

**S7. Limitations**

Outcomes from the index trial that are not recorded in routine care, namely the Patient-Specific Functional Scale, the global rating of change, the Fear-Avoidance Beliefs Questionnaire, and the deep neck flexor endurance test, could not be emulated. Routine outcome assessors were not blinded, and residual confounding from unmeasured factors such as pain beliefs and patient preference cannot be excluded.

**Supplementary Tables**

**Supplementary Table S1.** TARGET reporting checklist items and their location in this analysis report

| **TARGET item(s)** | **Description** | **Location in this report** |
| --- | --- | --- |
| **1a–c** | Abstract: emulation identified, data sources, key assumptions/findings | Summary of key results; Note on data |
| **2–4** | Background, causal question, rationale (cite index RCT) | Manuscript text; Table 1 |
| **5** | Data sources (purpose, type, setting, period, linkage) | Methods; supplementary table S2 |
| **6a–h** | Target trial specification | Table 1 (column 2) |
| **7a–h** | Emulation operationalisation | Table 1 (column 3); S2, S8–S10 |
| **8** | Participant selection / flow | Supplementary table S3 (flow) |
| **9** | Baseline data by strategy (± weighting) | Table 2 |
| **10** | Follow-up summary | Supplementary table S4 |
| **11** | Missing data by strategy | Supplementary table S4 |
| **12** | Outcome frequencies/distribution | Table 3; Table 4 |
| **13** | Effect estimates (absolute + relative) | Table 3; Table 4 |
| **14** | Additional/sensitivity analyses | Supplementary tables S5–S7 |
| **15–16** | Interpretation; limitations | Manuscript discussion |
| **17–21** | Ethics, registration, data sharing, funding, conflicts | Manuscript other information |

***Note:*** *The TARGET checklist comprises 21 items across six sections (abstract, introduction, methods, results, discussion, other information), with items 6a–h specifying the target trial and items 7a–h describing its emulation. This table maps each item (or item group) to the location in this report where it is addressed. Items 2–4 and 15–21 are addressed in the main manuscript text, which accompanies these tables.*
***Abbreviations:*** *EHR=electronic health record; ITT=intention-to-treat; TARGET=Transparent Reporting of Observational Studies Emulating a Target Trial.*

**Supplementary Table S2. Data sources, covariate definitions and measurement windows**

| **Domain** | **Source** | **Measurement window** | **Notes** |
| --- | --- | --- | --- |
| **Demographics** | Structured registration tables | At time zero | Age, sex; near-complete |
| **Diagnoses** | ICD-10 National Clinical Version 2.0 | ≤ time zero (lookback to all history for exclusions) | See S8 |
| **Procedures / exposures** | National medical-service price-item charges + free-text notes (NLP) | At and after time zero | See S9; dry needling vs acupuncture and manual therapy vs tuina disambiguated |
| **Medications** | Chinese generic name (通用名); ATC not used in participating hospitals | 12 months pre–time zero (baseline); after time zero (time-varying) | See S10 |
| **Outcomes (NDI, NPRS)** | Rehabilitation PRO tables / notes | Baseline; 2–3 wk; 4–6 wk; 3 mo (windowed) | Simplified-Chinese NDI |
| **Comorbidities** | ICD-10 + notes | 12 months pre–time zero | Depression, anxiety, fibromyalgia, Charlson |
| **Imaging** | Radiology report text/impression | ≤ time zero | Degenerative/disc findings |

***Note:*** *All eligibility and confounder variables were ascertained using only information available at or before time zero, except exclusion diagnoses (e.g. prior surgery, malignancy), for which the full available lookback was used. Time-varying co-interventions and interim outcomes were ascertained after time zero for the per-protocol analysis. Medications are stored by Chinese generic name in the participating hospital information systems; the Anatomical Therapeutic Chemical classification is not used. Detailed code lists appear in supplementary tables S8–S10.*
***Abbreviations:*** *ATC=Anatomical Therapeutic Chemical (classification); EHR=electronic health record; ICD-10=International Classification of Diseases, 10th Revision (China National Clinical Version 2.0); NDI=Neck Disability Index; NLP=natural language processing; NPRS=Numeric Pain Rating Scale; PRO=patient-reported outcome; TCM=traditional Chinese medicine; wk=week(s).*

**Supplementary Table S3. Participant flow and derivation of the analysis cohort**

| **Step** | **n** |
| --- | --- |
| Patients in screening pool (mechanical neck pain encounters, 2019–2024) | 7,373 |
| Excluded at screening (ineligible diagnosis, exclusions, prior DN/MT exposure, insufficient lookback) | 1,480 |
| Eligible new users initiating dry needling or manual therapy (analysis cohort) | 5,893 |
| Dry needling strategy | 1,995 |
| Manual therapy strategy | 3,898 |
| Adherent to assigned strategy (per-protocol eligible): dry needling | 356 / 1,995 |
| Adherent to assigned strategy (per-protocol eligible): manual therapy | 1,239 / 3,898 |
| With observed primary outcome (NDI at 3 months): dry needling | 584 |
| With observed primary outcome (NDI at 3 months): manual therapy | 1,928 |

***Note:*** *The screening pool comprises all patients with a mechanical neck pain encounter during the study period. Patients were excluded for ineligible diagnosis, the presence of any exclusion criterion, prior dry needling or manual therapy within the 12-month washout window, or insufficient pre–time-zero observation. Each eligible patient contributes a single time zero (first qualifying initiation episode). Per-protocol eligibility and observed-outcome counts are shown by treatment strategy; the denominators differ because of differential adherence and outcome ascertainment between groups.*
***Abbreviations:*** *DN=dry needling; MT=manual therapy; NDI=Neck Disability Index.*

**Supplementary Table S4. Missing data by treatment strategy and reasons for end of follow-up**

| **Variable** | **Dry needling % missing** | **Manual therapy % missing** | **Overall % missing** |
| --- | --- | --- | --- |
| Body mass index | 36.0 | 32.7 | 33.8 |
| Smoking status | 37.2 | 37.5 | 37.4 |
| Occupation | 42.3 | 43.2 | 42.9 |
| Symptom duration | 24.6 | 25.0 | 24.8 |
| Depression | 12.5 | 13.3 | 13.0 |
| NDI baseline | 8.7 | 7.3 | 7.8 |
| NPRS baseline | 7.9 | 8.1 | 8.0 |
| NDI 2–3 wk | 27.2 | 18.3 | 21.3 |
| NDI 4–6 wk | 52.6 | 35.1 | 41.0 |
| NDI 3 mo | 70.7 | 50.5 | 57.4 |
| NPRS 2–3 wk | 27.2 | 18.3 | 21.3 |
| NPRS 4–6 wk | 52.6 | 35.1 | 41.0 |
| NPRS 3 mo | 70.7 | 50.5 | 57.4 |

***Note:*** *Values are the percentage of records with a missing value for each variable, by treatment strategy and overall. Overall missingness across the dataset was 12.8%. Administrative variables (identifiers, age, sex, dates, eligibility flags) were essentially complete and are not shown. Outcome missingness increased monotonically over follow-up (2–3 weeks < 4–6 weeks < 3 months) and was higher in the dry-needling arm, in more severe patients and during periods of COVID-19 service disruption, consistent with a missing-at-random mechanism. Missing data were handled by multiple imputation by chained equations for the primary analyses. End-of-follow-up reasons: completed 3-month window, 3652 patients; loss to follow-up, 2151; administrative end (31 December 2024), 84; death, 6.*
***Abbreviations:*** *BMI=body mass index; COVID-19=coronavirus disease 2019; mo=month(s); NDI=Neck Disability Index; NPRS=Numeric Pain Rating Scale; wk=week(s).*

**Supplementary Table S5. Propensity-score overlap, weight distribution and balance diagnostics**

| **Diagnostic** | **Value** |
| --- | --- |
| Propensity-score range (all patients) | 0.030 to 0.970 |
| Median propensity score, dry needling | 0.473 |
| Median propensity score, manual therapy | 0.212 |
| Stabilized weight, mean | 0.99 |
| Stabilized weight, 99th percentile (truncation point) | 3.58 |
| Stabilized weight, maximum before truncation | 8.23 |
| Maximum \|SMD\| before weighting | 0.72 |
| Maximum \|SMD\| after weighting | 0.08 |
| Covariates with \|SMD\|<0.10 after weighting | 20/20 |

***Note:*** *Propensity scores (probability of receiving dry needling) were estimated by logistic regression on all baseline covariates listed in Table 2. Stabilized weights were truncated at the 1st and 99th percentiles. Propensity scores spanned the full (0,1) interval with substantial overlap between groups, supporting the positivity assumption. The stabilized weights were well-behaved (mean ≈1), and weighting reduced the maximum standardized mean difference from above 0.7 to below 0.10.*
***Abbreviations:*** *IPTW=inverse-probability-of-treatment weighting; PS=propensity score; SMD=standardized mean difference.*

**Supplementary Table S6. Sensitivity analyses for the primary outcome (NDI at 3 months, dry needling minus manual therapy)**

| **Analysis** | **Mean difference (95% CI)** | **Comment** |
| --- | --- | --- |
| Crude (unadjusted) | +10.1 (+9.4 to +10.7) | Confounded by indication |
| Stabilized IPTW (ITT-analogue) | +5.7 (+5.0 to +6.5) | Primary ITT-analogue |
| IPTW + baseline NDI adjustment | +5.7 (+5.2 to +6.2) | Doubly adjusted |
| Multivariable regression (no weighting) | +6.4 (+6.0 to +6.8) | Outcome-regression alternative |
| Clone–censor–weight (per-protocol) | +5.7 (+4.9 to +6.4) | Per-protocol effect (bootstrap CI) |
| Site 1 (IPTW) | +4.6 (+3.4 to +5.8) | n=957 |
| Site 2 (IPTW) | +6.3 (+5.0 to +7.6) | n=915 |
| Site 3 (IPTW) | +6.5 (+5.1 to +7.9) | n=640 |

***Note:*** *All estimates are mean differences in the 3-month NDI (dry needling minus manual therapy; positive = worse for dry needling). The crude estimate is unadjusted; the IPTW estimate is the primary intention-to-treat–analogue analysis; further rows add baseline-NDI adjustment to the weighted model, replace weighting with multivariable outcome regression, restrict to the per-protocol population via clone–censor–weighting, and repeat the IPTW analysis within each hospital site. All confounding-adjusted analyses converge near +5.7 to +6.4 points, recovering the embedded true effect (+6.5), whereas the crude estimate (+10.1) materially overstates the difference. Consistency across methods and sites supports the robustness of the adjusted estimate.*
***Symbols:*** *95% CIs are from robust (HC1) variance except the clone–censor–weight row (200 bootstrap resamples).*
***Abbreviations:*** *CCW=clone–censor–weight; CI=confidence interval; IPTW=inverse-probability-of-treatment weighting; ITT=intention-to-treat; NDI=Neck Disability Index.*

**Supplementary Table S7. Quantitative bias analysis: E-values and negative-control outcome**

| **Diagnostic** | **Value** | **Interpretation** |
| --- | --- | --- |
| E-value for the point estimate (primary IPTW) | 3.62 | Minimum joint confounder association (risk-ratio scale) needed to explain away the estimate |
| E-value for the CI limit closest to the null | 3.22 | Confounding strength needed to shift the CI to include no effect |
| Negative-control outcome (weighted DN−MT) | +0.001 (-0.030 to +0.031) | Association near null as expected; no residual systematic bias detected |

***Note:*** *The E-value is the minimum strength of association, on the risk-ratio scale, that an unmeasured confounder would need to have with both the treatment and the outcome—above and beyond the measured covariates—to fully explain away the observed effect. Larger E-values indicate greater robustness to unmeasured confounding. The continuous mean difference was converted to an approximate risk-ratio scale before computing the E-value. The negative-control outcome (a baseline indicator with no plausible causal link to treatment) showed no association with treatment after weighting, providing no evidence of residual systematic bias.*
***Abbreviations:*** *CI=confidence interval; DN=dry needling; MT=manual therapy.*

**Supplementary Table S8. Diagnosis code list (ICD-10 National Clinical Version 2.0)**

| **ICD-10 code** | **English term** | **Role in study** |
| --- | --- | --- |
| **M54.2** | Cervicalgia (mechanical neck pain) | Inclusion (index condition) |
| **M53.1** | Cervicobrachial syndrome | Inclusion / covariate |
| **M47.8** | Spondylosis NOS (cervical spondylosis, non-myelopathic subtypes) | Inclusion |
| **M47.2 (+G55.2*)** | Spondylosis with radiculopathy | Covariate |
| **M47.1 (+G99.2*)** | Spondylosis with myelopathy | Exclusion |
| **M50.0 (+G99.2*)** | Cervical disc disorder with myelopathy | Exclusion |
| **M50.1 (+G55.1*)** | Cervical disc disorder with radiculopathy | Exclusion / covariate |
| **M50.3** | Other cervical disc degeneration | Inclusion / covariate |
| **S13.4** | Cervical sprain / whiplash | Exclusion (within 6 weeks) |
| **S12** | Fracture of cervical vertebra | Exclusion (red flag) |
| **C00–C97 / C79.5** | Malignancy / bone metastasis | Exclusion (red flag) |
| **M46.2–M46.5** | Vertebral osteomyelitis / discitis / spinal infection | Exclusion (red flag) |
| **M05 / M06** | Rheumatoid arthritis | Exclusion / covariate |
| **M45** | Ankylosing spondylitis | Exclusion / covariate |
| **D68** | Coagulopathy | Exclusion (dry-needling contraindication) |
| **F32 / F33** | Depressive episode / recurrent depression | Covariate |
| **F40 / F41** | Phobic / other anxiety disorders | Covariate |
| **M79.7** | Fibromyalgia | Covariate |

***Note:*** *Diagnoses were identified from the structured diagnosis tables using ICD-10 codes (China National Clinical Version 2.0) and confirmed, where required, by free-text review. Codes serve as inclusion criteria (index condition), exclusion criteria (red flags and contraindications) or baseline covariates, as indicated. Cervical spondylosis subtypes map to M47.8 unless myelopathy or radiculopathy is documented, in which case the corresponding manifestation code is added.*
***Symbols:*** *An asterisk (*) denotes an etiology/manifestation (dagger–asterisk) code applied per Chinese coding convention (e.g. G99.2*, G55.x*).*
***Abbreviations:*** *ICD-10=International Classification of Diseases, 10th Revision; NOS=not otherwise specified.*

**Supplementary Table S9. Procedure / charge-item code list (national medical-service price items) and free-text terms**

| **Item** | **Item code / category** | **Role in study** |
| --- | --- | --- |
| Dry needling / trigger-point needling | NA | Exposure: dry needling |
| Body / electro- / warm acupuncture | NA | Distinguished from dry needling by note review |
| Tuina / cervical-region tuina | NA | Distinguished from manual therapy |
| Manual therapy / spinal manipulation | NA | Exposure: manual therapy (manipulation) |
| Joint mobilisation | 340200026 | Exposure: manual therapy (mobilisation) |
| Therapeutic exercise | 340200020 | Co-intervention (both arms) |
| Cervical traction | 340200xxx | Co-intervention / covariate |
| Low-/medium-frequency electrotherapy | 340000xxx | Co-intervention |
| Therapeutic ultrasound | 340000xxx | Co-intervention |
| Infrared / herbal hot-compress therapy | 340000xxx | Co-intervention |

***Note:*** *Procedures were identified from structured charge/price-item records and free-text rehabilitation notes. Dry needling was distinguished from traditional acupuncture, and manual therapy from tuina/massage, by combining item codes with natural-language-processing of the procedure description and dual independent chart review for validation. Item codes reflect the national medical-service price-item specification; the 2024–2025 (item-listing guidance) consolidated several traditional Chinese medicine items into broader categories. Exact operative codes are assigned by each hospital's provincial price file.*
***Abbreviations:*** *NLP=natural language processing; TCM=traditional Chinese medicine; xxx=placeholder for hospital/province-specific code digits.*

**Supplementary Table S10. Medication list (recorded by Chinese generic name; ATC not used in participating hospitals)**

| **Generic name** | **Class** | **Role in study** |
| --- | --- | --- |
| Ibuprofen | NSAID | Baseline covariate / time-varying co-intervention |
| Diclofenac sodium | NSAID | Covariate / co-intervention |
| Celecoxib | COX-2 inhibitor | Covariate / co-intervention |
| Loxoprofen sodium | NSAID | Covariate / co-intervention |
| Meloxicam | NSAID | Covariate / co-intervention |
| Etoricoxib | COX-2 inhibitor | Covariate / co-intervention |
| Naproxen | NSAID | Covariate / co-intervention |
| Indomethacin | NSAID | Covariate / co-intervention |
| Flurbiprofen patch | Topical NSAID | Covariate / co-intervention |
| Diclofenac diethylamine emulgel | Topical NSAID | Covariate / co-intervention |
| Paracetamol | Non-opioid analgesic | Covariate / co-intervention |
| Tramadol | Opioid | Covariate (severity marker) |
| Oxycodone(-paracetamol) | Opioid | Covariate (severity marker) |
| Codeine | Opioid | Covariate |
| Eperisone | Muscle relaxant | Covariate / co-intervention |
| Chlorzoxazone | Muscle relaxant | Covariate / co-intervention |
| Tizanidine | Muscle relaxant | Covariate / co-intervention |
| Baclofen | Muscle relaxant | Covariate |
| Amitriptyline | Tricyclic antidepressant | Covariate |
| Duloxetine | SNRI | Covariate |
| Venlafaxine | SNRI | Covariate |
| Sertraline / fluoxetine / paroxetine / escitalopram | SSRI | Covariate |
| Pregabalin / gabapentin | Neuropathic agent | Covariate |
| Mecobalamin | Neurotrophic | Covariate |
| Warfarin | Anticoagulant | Contraindication exclusion (dry needling) |
| Rivaroxaban / apixaban / dabigatran | Direct oral anticoagulant | Contraindication exclusion |
| Enoxaparin / nadroparin / dalteparin | Low-molecular-weight heparin | Contraindication exclusion |

***Note:*** *Medications were identified by Chinese generic name as stored in the hospital information systems; the participating hospitals do not record Anatomical Therapeutic Chemical (ATC) codes. Anticoagulants (warfarin, direct oral anticoagulants and low-molecular-weight heparins) define a contraindication exclusion for dry needling. Analgesics, muscle relaxants, antidepressants and neuropathic agents are baseline confounders ascertained in the 12 months before time zero and, where initiated or changed after time zero, time-varying co-interventions in the per-protocol analysis. Where several agents of one class are listed in a single row, any one qualifies.*
***Abbreviations:*** *ATC=Anatomical Therapeutic Chemical (classification); COX-2=cyclo-oxygenase-2; NSAID=non-steroidal anti-inflammatory drug; SNRI=serotonin–noradrenaline reuptake inhibitor; SSRI=selective serotonin reuptake inhibitor.*

**Supplementary Table S11. Validation of the exposure-classification algorithm against blinded manual chart review**

| **Metric** | **Dry needling** | **Manual therapy** | **Overall** |
| --- | --- | --- | --- |
| Episodes adjudicated on chart review, n | 1772 | 3505 | 5277 |
| Confirmed correct on chart review, n | 1625 | 3197 | 4822 |
| Misclassified (acupuncture or tuina/massage), n | 147 | 308 | 455 |
| Positive predictive value, % | 91.7 | 91.2 | 91.4 |
| Observed agreement, % | — | — | 91.4 |
| Cohen’s κ (95% interpretation) | — | — | 0.81 (substantial) |
| Indeterminate on review, n (%) | 223 (11.2) | 393 (10.1) | 616 (10.5) |

***Note:*** *An independent reviewer, blinded to the algorithm output, adjudicated the treatment actually delivered in each episode (the reference standard). Ascertainment sources were: structured code only, 1535 episodes; free-text or natural-language processing only, 885; both code and text, 2610; and chart-abstracted, 863. The positive predictive value is the proportion of algorithm-assigned episodes confirmed correct on review. Because the reference standard was applied only to episodes already classified as dry needling or manual therapy, classical sensitivity and specificity are not identifiable; the positive predictive value and Cohen’s κ are the appropriate metrics. Indeterminate episodes were retained in the primary analysis and removed in a sensitivity analysis.*

**Supplementary Table S12. Complete-case versus multiply-imputed primary analysis and missing-not-at-random (pattern-mixture) sensitivity analysis**

| **Analysis** | **3-month NDI difference (95% CI)** | **n** |
| --- | --- | --- |
| Complete-case (weighted) | 5.7 (5.0 to 6.5) | 2512 |
| Multiple imputation (20 datasets, weighted) | 6.2 (5.8 to 6.7) | 5893 |

*Part A. The complete-case and imputed estimates agree closely. The fraction of missing information for the imputed primary estimate was 14%. Positive values indicate greater improvement (lower disability) with manual therapy (dry needling minus manual therapy).*

| **δ applied to unobserved dry-needling outcomes (NDI points)** | **3-month NDI difference (95% CI)** |
| --- | --- |
| -4 | +3.49 (+3.06 to +3.92) |
| -3 | +4.16 (+3.73 to +4.59) |
| -2 | +4.83 (+4.40 to +5.25) |
| -1 | +5.49 (+5.06 to +5.92) |
| +0 | +6.16 (+5.73 to +6.59) |
| +1 | +6.83 (+6.39 to +7.27) |
| +2 | +7.50 (+7.06 to +7.94) |
| +3 | +8.17 (+7.72 to +8.61) |
| +4 | +8.83 (+8.38 to +9.29) |

*Part B. Missing-not-at-random pattern-mixture (δ-adjustment) sensitivity analysis. Imputed 3-month Neck Disability Index values in the dry-needling arm were shifted by δ points before re-estimating the weighted difference. Positive δ assumes unobserved dry-needling patients had worse (higher) disability than imputed; negative δ assumes better (lower) disability. The manual-therapy advantage persists across the entire range: even at δ = −4, the adjusted difference remains 3.5 Neck Disability Index points in favour of manual therapy. Positive values indicate greater improvement with manual therapy (dry needling minus manual therapy).*

**Supplementary figures**

Supplementary Figure S1.

Propensity-score distribution and overlap by treatment strategy. The mirrored histogram shows the estimated probability of dry needling in each group. Overlap across the full range supports the positivity assumption.

Supplementary Figure S2.

Distribution of stabilized inverse probability of treatment weights by treatment strategy. The dashed line marks the 99th centile, at which weights were truncated.

Supplementary Figure S3.

Effect of dry needling versus manual therapy on the 3-month NPRS across analyses. Points and horizontal lines show mean differences with 95% confidence intervals.
